# C23 promotes tumorigenesis via suppressing p53 activity

**DOI:** 10.18632/oncotarget.11071

**Published:** 2016-08-05

**Authors:** Qun Li, Yan Zhu, Lili Hou, Juan Wang, Guilin Hu, Xing Fang, Yamin Hu, Tingting Tao, Xin Wei, Haitao Tang, Baojun Huang, Wanglai Hu

**Affiliations:** ^1^ Department of Immunology, Anhui Medical University, Hefei, China; ^2^ Department of Anesthesiology, The First Affiliated Hospital of Anhui Medical University, Hefei, China; ^3^ Department of Clinical Nutriology, The First Affiliated Hospital of Anhui Medical University, Hefei, China; ^4^ Department of Molecular Biology, Shanxi Cancer Hospital and Institute, Affiliated Hospital of Shanxi Medical University, Taiyuan, China; ^5^ School of Life Sciences, Anhui Medical University, Hefei, China; ^6^ School of Pharmacy, Anhui Medical University, Hefei, China

**Keywords:** C23, p53, tumorigenesis

## Abstract

C23 is an abundant and multi-functional protein, which plays an important role in various biological processes, including ribosome biogenesis and maturation, cell cycle checkpoints and transcriptional regulation [1, 2]. However, the role of C23 in controlling tumorigenesis has not been well defined. Here we report that C23 is highly expressed in cancer cells and the elevated expression of C23 facilitates cancer cell proliferation *in vitro* and tumor xenograft growth *in vivo*. Notably, C23 binds to p53 through its GAR domain and suppresses the transcriptional activity of p53 under DNA damage and hypoxia. Moreover, the GAR domain is critical for C23-mediated tumor cell proliferation both *in vitro* and *in vivo*. Our findings reveal a novel role of C23 in tumorigenesis and suggest that C23 may represent a potential therapeutic target for treating malignancy.

## INTRODUCTION

p53, the most renowned tumor suppressor, is activated in response to a wide range of cellular stress including DNA damage, oncogene activation and hypoxia [3–5]. The activated p53 prevents tumor development by invokes anti-proliferative processes, of which the best established include apoptotic cell death, cell cycle arrest [6–9]. The potent effect of p53 on tumor formation makes its strict regulation as a central issue in human cells. In human cancers, to permit cellular survival and proliferation, the normal p53 signal pathway is mainly impaired in two patterns [8]. First, the gene encoding p53 undergoes inactivating mutations in >50% of human cancers and mutations≥ 18,000 in many different cancers. These mutations are mainly found in the core region of p53 DNA binding domain (residues 98-292), rendering tumor genesis [10, 11]. In addition, the full-length mutant p53 proteins are accumulated in many types of cancer. The second, in cancers harboring wild-type p53, the activity of p53 is frequently inhibited by a range of different mechanisms [12, 13]. p53 protein often forms a complex with its partners those modulated the activity of p53 [14]. Particularly, two ASPP protein family members, ASPP1 and ASPP2, bind to the DNA binding domain of p53 and enhance apoptosis triggered by DNA damage [15]. The DNA binding protein YB1 directly binds to p53 with UV irradiation, preventing transactivation of proapoptotic genes [16]. The p53 family members p63 and p73, similarly modulate p53 activity through binding to p53 [17]. Given the diversity and complexity of the binding partners of p53, discovering the underlying collaborators and/or regulators of p53 is still a priority for fully understanding the tumor suppressive role of p53.

C23, also called nucleolin, is a c-myc-responsive gene [18, 19]. C23 plays an essential role in ribosome biogenesis including rRNA synthesis, processing and assembly of precursor ribosomal RNA (pre-rRNA), and transport of ribosomal proteins out of the nucleus [18, 20]. In addition, C23 interacts with some mRNA molecules, including the apoptosis inhibitor BCL-2 with taxol or okadaic acid treatment [21] and the cell-cycle regulator GADD45A under oxidative stress [22], to regulate the turnover rate of these mRNAs. In response to heat shock or genotoxic stress, C23 blocks the replication initiation of chromosomal DNA by binding and repressing the cellular single-stranded DNA binding protein RPA (replication protein A) [23, 24]. Moreover, C23 and rad51 complex plays an important role in DNA repair triggered by homologous recombination [25]. Of note, C23 contributes to the maintenance of telomere via binding to the catalytic subunit of human telomerase reverse transcriptase (hTERT) [26]. Recently, increasing evidences indicate the association between C23 and p53. For instance, C23 and ribosomal protein L26 directly regulates the translation and induction of p53 upon DNA damage [27]. Genotoxic stress mobilizes C23-p53 complex formation, leading to inhibition of transient replication and DNA repair [28]. Interestingly, C23 stabilizes p53 through inhibiting mdm2 in response to hyperproliferative signals [29]. Furthermore, C23 maintains embryonic stem cell self-renewal by suppressing a p53-dependent signal pathway [30]. However, the functional consequence of C23 in tumorigenesis via suppression of p53 is still poorly understood.

In this study, we report that C23 plays a critical role in tumorigenesis, in which, the glycine/arginine-rich (GAR) domain of C23 is required. Moreover, we demonstrate that C23 binds to p53 through its GAR domain and thus suppresses its transcriptional activity under hypoxia and DNA damage condition. Of note, the vast majority of cancer lesions express elevated levels of C23 compared to the corresponding normal adjacent tissues. Collectively, we propose that C23 is an important regulator in tumorigenesis.

## RESULTS

### Elevated expression levels of C23 in cancer cells

**Figure 1:**
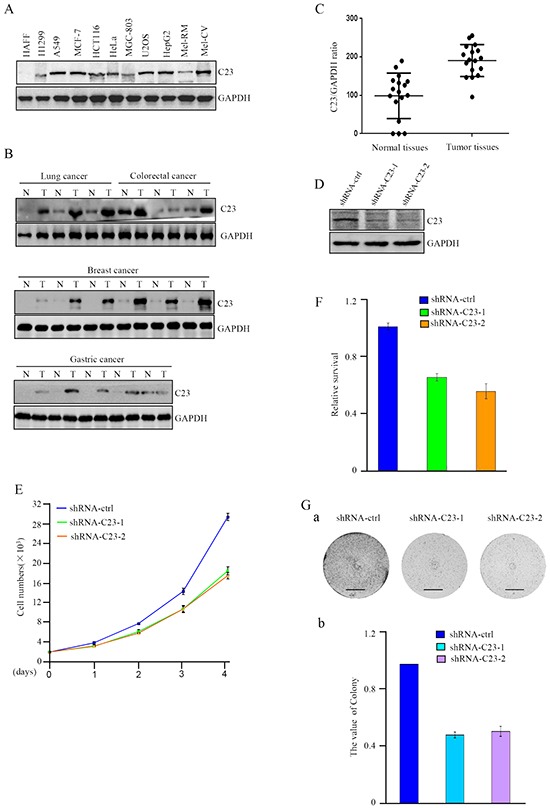
C23 expression levels were elevated in cancer cells and critical for cancer cell proliferation **A.** Protein level of C23 was determined by western blot in different cancer cell lines. GAPDH was used as loading control. **B-C.** C23 expression levels in human cancer lesions and corresponding normal adjacent tissues were analyzed by Western blot analysis (B) and Graphpad prism (C). **D.** C23 protein levels were evaluated by western blot in HCT116 cells stably expressing the control shRNA, C23 shRNA1 or C23 shRNA2. GAPDH served as loading control. **E.** The number of HCT116 cells stably expressing the control shRNA, C23 shRNA1 or C23 shRNA2 was determined by cell counter. The data were represented as mean±S.D. of three independent experiments. **F.** HCT116 cells stably expressing the control shRNA, C23 shRNA1 or C23 shRNA2 were treated with 100ng/ml Doxorubicin for 24 h. Viability of cells was determined using MTT assays by measuring the absorbance at 490 nm in a microplate reader. **G.** Long-term colony formation assay of HCT116 cells with and without stable knockdown of C23. Cells (5000 cells per well) were allowed to grow for 3 weeks, then stained and photographed. Scale bar, 1cm.

Previous studies suggested that C23 may play a critical role in tumor progression. In this study, we examined the expression levels of C23 in a panel of cancer cell lines, including lung cancer (H1299, A549), colorectal cancer (HCT116), breast cancer (MCF-7), gastric cancer (MGC-803), osteosarcoma (U2OS), hepatocellular carcinoma(HepG2) and melanoma (Mel-CV and Mel-RM). The results revealed that the C23 expression levels were frequently upregulated in cancer cell lines in comparison with the normal cell line (human adult foreskin fibroblast, HAFF) (Figure 1A). To further confirm this observation, we analyzed 17 paired human cancer lesions and corresponding normal adjacent tissues by use of Western blot and Graphpad prism and found that cancer lesions displayed relatively higher levels of C23 in comparison with normal adjacent tissues (Figure 1B and 1C).

### C23 is critical for cancer cell proliferation

To determine the physiological and pathological consequences of highly expressed C23 in cancer cells, we established HCT116 sub cell line stably expressing short hairpin RNAs (shRNAs) targeting C23 (Figure 1D), and evaluated the effect of C23 on cancer cell proliferation. As shown in Figure 1E and 1F, the proliferation and viability of cancer cells were significantly impaired without C23. Furthermore, the effect of C23 on cancer cell proliferation was confirmed by long-term colony formation assay (Figure 1Ga and 1Gb). Taken together, the data demonstrated that C23 was highly expressed in cancer lesions and cancer cell lines, which may indicate the important role of C23 in human tumor progression.

### C23 interacts with p53 through GAR domain

**Figure 2:**
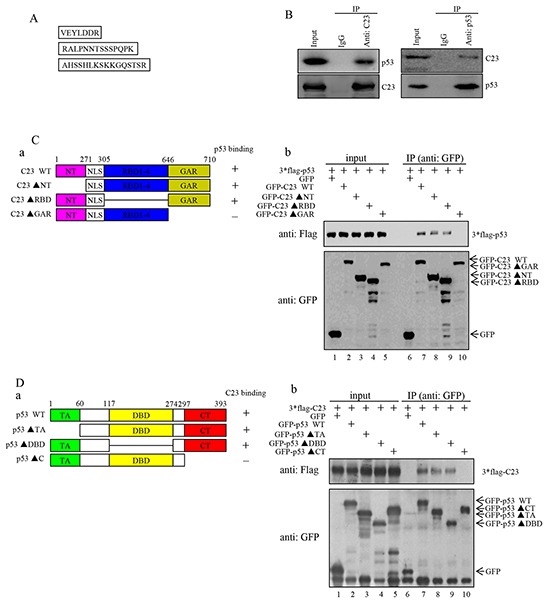

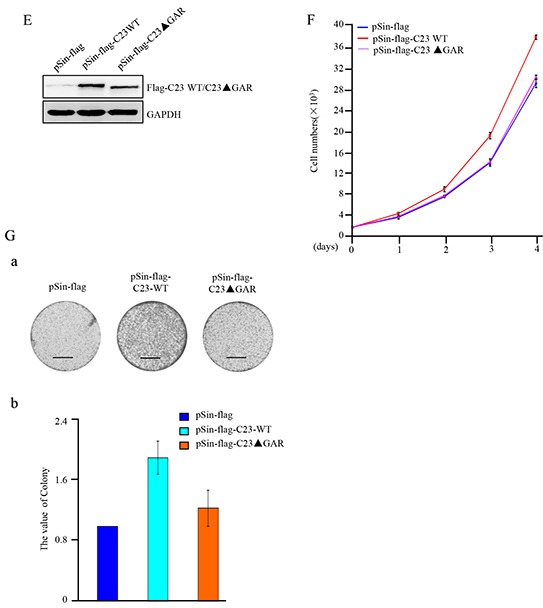
C23 bound to p53 through the GAR domain which was required for C23-mediated cancer cell proliferation **A.** HCT116 cell lysates were incubated with anti-IgG and anti-C23 coupled to Protein A/G-Sepharose beads. The bead-bound proteins were boiled in SDS sample buffer and eluted proteins were subjected to mass spectrometry analysis. Three peptides obtained by LC-MS were found to match the tumor protein p53 (TP53). **B.** The binding between endogenous C23 and p53. (Left) Lysates from HCT116 (p53+/+) cells were immunoprecipitated with anti-C23 or anti-IgG (anti-mouse). (Right) Lysates from HCT116 cells were immunoprecipitated using anti-p53 or anti-IgG (anti-rabbit). Both immunoprecipitates were analyzed by western blot analysis with the indicated antibodies. **C.** (a) The GFP-fused C23 and the corresponding deletion mutants were schematic illustrated. NT, N-terminal domain; RBD, RNA-binding domain; GAR, glycine-arginine rich domain; NLS, nuclear localization sequences. The amino acid residues at the domain boundaries were indicated. The binding of C23 mutants to p53 was indicated by a plus sign, and the lack of binding was indicated with a minus sign. (b) GFP-fused C23 and the corresponding deletion mutants were individually co-transfected with 3×Flag-p53 into HEK 293T cells. 24 hours after transfection, cells were treated with 25 mM MG132 for an additional 6 hours. The cell lysates were then immunoprecipitated with anti-GFP antibody and subjected to Western blot analysis. **D.** (a) A schematic representation of p53 and the corresponding mutants. TA, transcriptional activation domain; DBD, DNA binding domain; CT, C-terminal domain. (b) GFP-tagged p53 and the corresponding deletion mutants were individually co-transfected with 3*Flag-C23 in HEK 293T cells. 24 hours after transfection, cells were treated with 25 mM MG132 for an additional 6 hours. The cell lysates were then subjected to immunoprecipitation with anti-GFP and subsequent Western blot analysis. **E.** HCT116 cells forced expressing C23 and C23ΔGAR were analyzed with anti-flag antibody. **F.** The number of HCT116 cells stably expressing C23 and C23ΔGAR were analyzed by cell counter. The data were represented as mean±S.D. of three independent experiments. **G.** HCT116 cells stably expressing C23 and C23ΔGAR were seeded onto six-well plates (5000 cells per well) and were allowed to grow for 3 weeks before being fixed with methanol and stained with crystal violet. Scale bar, 1cm.

In order to investigate the mechanism of how C23 modulates the proliferation of cancer cells, we performed co-immunoprecipitation assay and subsequent mass spectrometry analysis using the lysates from HCT116 cells with anti-IgG or anti-C23. Three peptides obtained by LC-MS were found to match tumor protein p53 (TP53) (Figure 2A). The results were further confirmed by reciprocal immunoprecipitation assays. As shown in Figure 2B, C23 and p53 were found to interact with each other directly under physiological conditions.

To delineate the regions of C23 that are responsible for its interaction with p53, we generated a panel of C23 deletion mutant constructs that fused to the GFP (Figure 2Ca) [31]. These mutant constructs were individually co-transfected into HEK 293T cells together with 3×Flag-p53. A co-immunoprecipitation assay was then performed to identify the regions of C23 protein responsible for binding to p53. As shown in Figure 2Cb, both C23 lacking the N-terminal domain(C23ΔNT) and C23 lacking the RNA-binding domains (C23ΔRBD1-4) were able to interact with p53 protein, while C23 lacking the glycine-arginine rich domain(C23ΔGAR) showed no interaction, thereby suggesting that the GAR domain of C23 is essential for its binding to p53. Similarly, we also generated a series of p53 deletion mutants and found that deletion of the transcriptional activation domain (p53ΔTA) and the DNA binding domain(p53ΔDBD) did not affect the p53-C23 interaction, while removal of C-terminal domain abolished the ability of p53 binding to C23 (Figure 2D). These data indicate that the C-terminal region of p53 and the GAR domain of C23 mediate the interaction between these two proteins.

### The GAR domain is essential for C23-mediated cancer cell proliferation

We next determined whether the GAR domain was involved in the cancer cell proliferation regulated by C23. HCT116 cell sublines stably overexpressing C23 or C23ΔGAR were established as shown in Figure 2E. C23 significantly enhanced the proliferation of HCT116 cells, which was abolished by the deletion of GAR domain (Figure 2F). Furthermore, long-term colony formation assay also revealed that deletion of the GAR domain of C23 lost the capability of C23 in regulation of cell proliferation (Figure 2Ga and 2Gb). Collectively, these results strongly suggest that the GAR domain of C23 is essential for C23-mediated cancer cell proliferation.

### C23 suppresses the transcriptional activity of p53 under hypoxia and DNA damage

**Figure 3:**
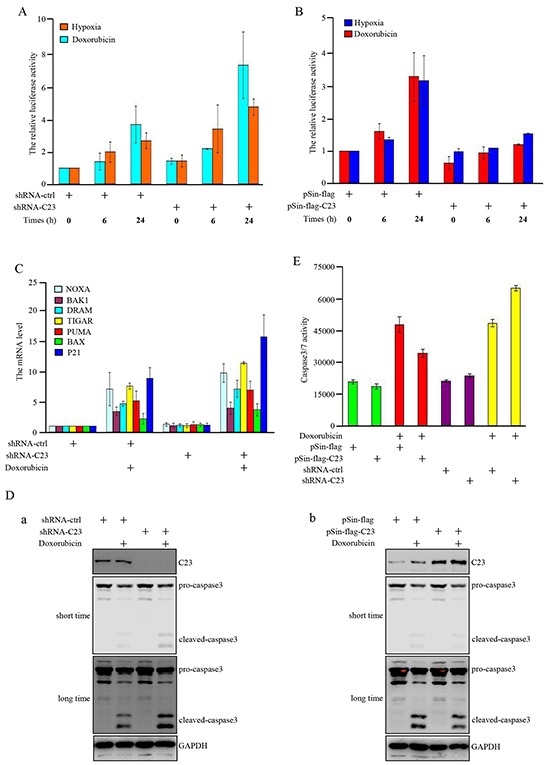

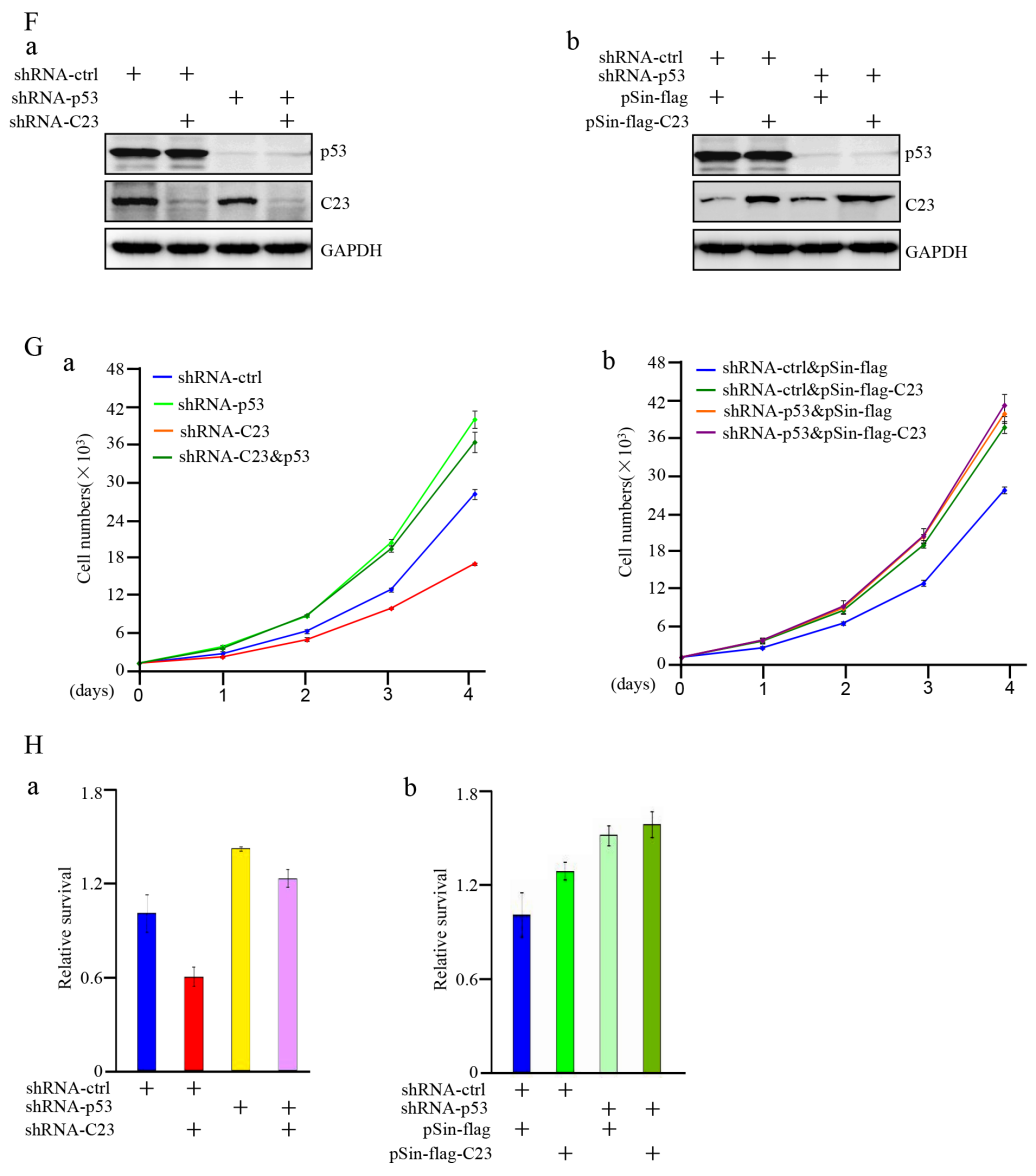
C23 suppressed the transcriptional activity of p53 upon DNA damage and hypoxia **A.** pGL3-3×p53-BS-LUC and Renilla were co-transfected in HCT116 cells combined with and without C23 shRNA. 24 hours after transfection, cells were treated with Doxorubicin (100ng/ml) or upon 1% O_2_ for indicated times and the cell lysates were analyzed by lucifease assay. **B.** HCT116 cells with and without stable overexpressing C23 were co-transfected with pGL3-3×p53-BS-LUC and Renilla. 24 hours after transfection, cells were treated with Doxorubicin (100ng/ml) or upon 1% O_2_ for indicated times and then the cell lysates were subjected to lucifease assays. **C.** HCT116 cells with and without stable knockdown of C23 were treated with Doxorubicin (100ng/ml) for 24 hours, then p53 target gene mRNA expression levels were analyzed by qRT-PCR analysis. **D-E.** HCT116 cells with and without stable overexpressing C23 (a) or stable knockdown of C23(b) were treated with Doxorubicin (100ng/ml) or mock control for 24 hours. Cell lysates were then subjected to Western blot analysis with the indicated antibodies (D) and caspase3/7 activity analysis (E), respectively. **F.** (a) HCT116 cells stably expressing the control shRNA, C23 shRNA, p53 shRNA,or C23 shRNA plus p53 shRNA. (b) HCT116 cells with and without p53 knockdown were introduced with C23 respectively. Protein level of C23 and p53 were evaluated by Western blot. GAPDH served as loading control. **G.** HCT116 cells stably expressing the control shRNA, C23 shRNA, p53 shRNA,or C23 shRNA plus p53 shRNA (a) and HCT116 cells stably knocked down p53 were additionally introduced with C23 respectively (b). Cell number was evaluated by cell counter. The data were represented as mean±S.D. from three independent experiments. **H.** (a) HCT116 cells stably expressing the control shRNA, C23 shRNA, p53 shRNA,or C23 shRNA plus p53 shRNA. (b) HCT116 cells stably knocked down p53 were co-transfected with C23 cDNA respectively. Cells were then treated with 100ng/ml Doxorubicin for 24 hours. Cell viability was determined by MTT assay. The data were shown as the mean±s.d. of three independent experiments.

Subsequently, we investigated how C23 contributes to cancer cell proliferation. It is widely accepted that p53 function as a tumor suppressor by being activated as a transcriptional factor, several p53 regulated genes were induced to prevent malignant transformation through prompting cell-cycle arrest and apoptosis [32, 33]. Therefore, we examined whether C23-p53 interaction could suppress the transcriptional activity of p53 and promote cell proliferation. Luciferase reporter assay revealed that C23 knockdown further enhanced the transcriptional activity of p53 under hypoxia condition and DNA damage drug treatment (Figure 3A). In contrast, the increase of transcriptional activity of p53 was reduced in cells with C23 cDNA overexpression (Figure 3B). Consistently, the expression levels of p53 target genes were additionaly up-regulated under hypoxia condition and DNA damage drug treatment when C23 was absence (Figure 3C, Supplementary Figure S1 and S2). In addition, as shown in Figure 3Da, knockdown of C23 obviously enhanced caspase-3 activation as shown in cleaved caspase-3. On the other hand, introduction of C23 cDNA into HCT116 cells resulted in a great decrease of caspase-3 activation under the same condition (Figure 3Db). Furthermore, the caspase3/7 activity analysis also revealed that C23 absence resulted in a dramatic increase in caspase3/7 activity in HCT116 cells after Doxorubicin treatment. Meanwhile, the caspase3/7 activity was reduced when C23 was overexpressed in cells (Figure 3E).

We further explored whether p53 was involved in C23-regulated cell proliferation. First, we established stable cell subline with knockdown of p53 in combination with C23 knockdown or overexpression (Figure 3F). Knockdown of p53 almost completely reversed the inhibitory effect of C23 knockdown on cell proliferation (Figure 3Ga). Similarly, the promoting effect of C23 overexpression on cell proliferation was abolished in HCT116 cells with p53 knockdown (Figure 3Gb). Next, we evaluated the effect of C23 on cell proliferation in the p53 null cell, the promoting effect of C23 overexpression on cell proliferation was significantly impaired in H1299 cells (Supplementary Figure S3). To further examine the impact of C23 on cell proliferation is dependent on p53 transactivation, pifithrin-alpha (PFT-α), an inhibitor of p53-dependent transcription was used. The impact of C23 on p53 transcriptional activity and cell proliferation was impeded under PFT-α treatment (Supplementary Figure S4). Furthermore, C23 protected cancer cells via p53 under Doxorubicin treatment as shown in Figure 3H. HCT116 cell subline harboring C23 shRNA exhibited a significant decrease in cell viability, however, the effect was extinct when p53 expression was reduced by shRNA (Figure 3Ha). Similarly, introduction of C23 cDNA resulted in an increase of cell viability which was reversed by knockdown of p53. Collectively, these results suggest that C23 can suppress the transcriptional activity of p53 under hypoxia and Doxorubicin treatment and p53 is involved in C23-promoted cell proliferation.

### C23 overexpression accelerates tumor growth in xenograft mouse model

**Figure 4:**
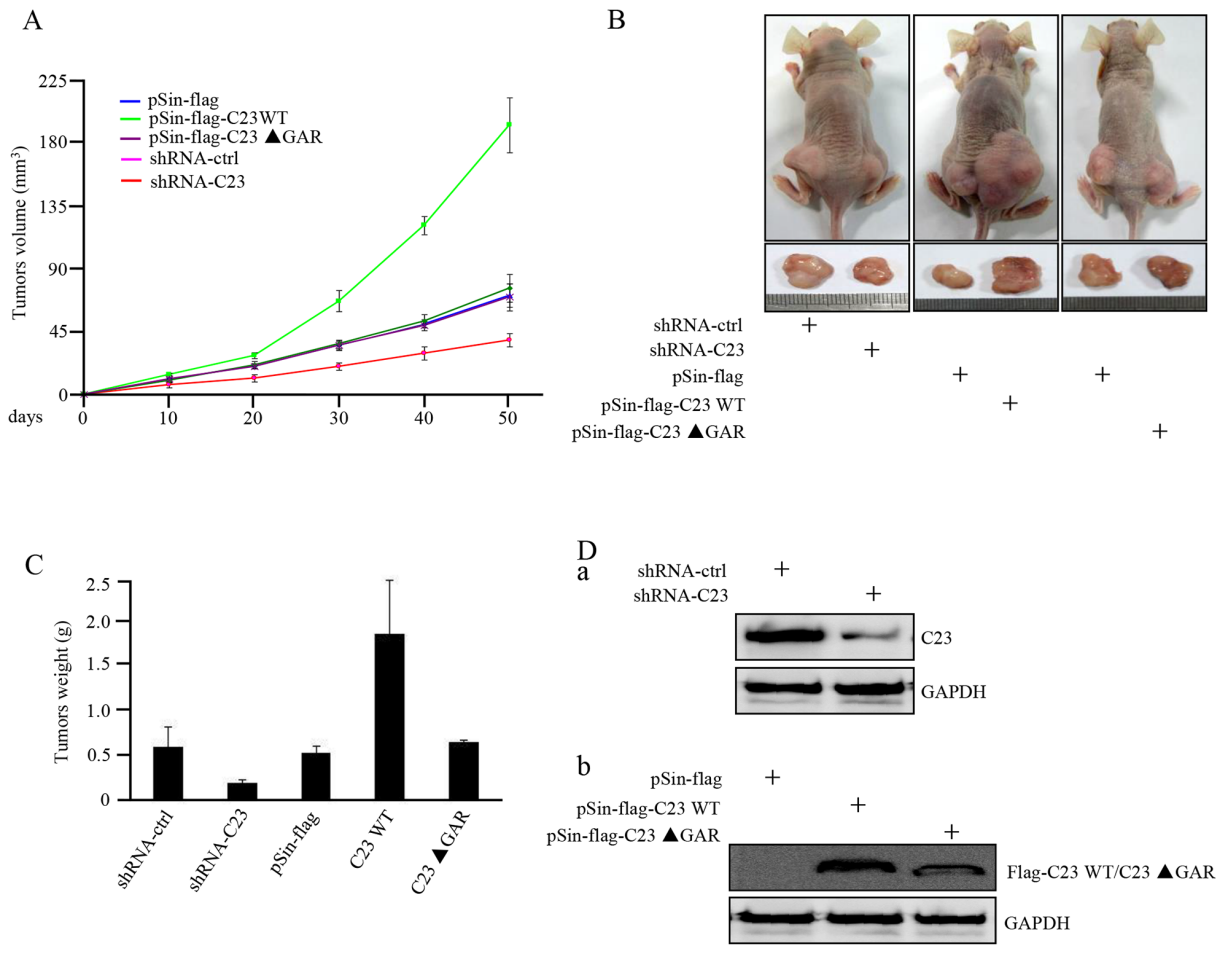
C23 overexpression increased tumor growth in xenograft mouse model **A.** HCT116 cells with and without stable knockdown of C23 or overexpression of wild-type C23 or C23 ΔGAR were individually injected subcutaneously into each flank of nude mice (n=6). Growth curves of tumor xenograft were evaluated via calculating the volumes of tumor. **B.** Representative photographs of tumor-bearing nude mice and tumors excised from the indicated mice were shown. **C.** Weights of the indicated tumors excised from nude mice. **D.** Whole-cell lysates from tumors excised from the indicated mice were subjected to Western blot analysis with the indicated antibodies.

The functional significance of C23 in tumor growth was further determined by use of xenograft mouse model. As shown in Figure 4A, deficiency in C23 resulted in marked retardation of tumor growth *in vivo*. In contrast, the cells stably overexpressing full length C23, but not C23ΔGAR, significantly enhanced the tumor growth. HCT116 cells with C23 stable knockdown formed smaller tumors than the corresponding control, and the tumors overexpessing C23 were larger in comparison with tumors overexpressing the vector control or C23ΔGAR (Figure 4B). This was further confirmed by measuring the xenograft weight (Figure 4C). Knockdown of C23, and overexpression of C23 and C23ΔGAR, were confirmed in tumor xenograft by use of Western blot analysis (Figure 4D). These results point to an important role of C23, particularly its GAR domain, in promoting tumor growth *in vivo*.

## DISCUSSION

Widely accepted concept is that p53 as a transcription factor plays a tumor suppressive role through mediating cell cycle arrest and apoptosis [32, 34]. However, the mechanisms of regulating p53 transcriptional activity remain largely unknown. In the present study, we demonstrated a novel mechanism that the elevated expression of C23 protein contributed to tumorigenesis via suppressing the transcriptional activity of p53.

C23 was originally identified as a c-myc-inducible protein that has been demonstrated to participate in various biological processes, including ribosome biogenesis, transportation of ribosomal proteins and many other cellular activities [1, 35]. However, whether C23 is involved in regulation of tumorigenesis remains uncharacterized. Current numerous studies identify C23 is essential for tumor proliferation and cell growth in many type of cells indicating that C23 may be a critical regulator in tumor formation [2, 36, 37]. Our data showed that C23 was highly expressed in the vast majority of tumor cell lines and cancer tissues, and inhibition of C23 expression decreased the tumor cell proliferation both *in vitro* and *in vivo*.

Even though the p53 can be function in a transcription-independent manner, more often, p53 exerts its tumor suppressor activity in a transcription-dependent manner [33]. Numerous studies have suggested that the transcriptional activity of p53 was precisely regulated in human tumors, the protein expression level of p53 itself and p53 binding parterners have been implicated in modulating the p53 transcriptional response [12, 14]. A recent report has shown that p53 expression level was further upregulated upon DNA damage by inhibition of C23 in HeLa, MCF-7 and primary fibroblast cell lines. The increased level of p53 was due to the enhanced mRNA stability of p53 regulated by C23 [27, 38]. It was also reported that knockdown of C23 elevated p53 through increasing its protein stability directly in embryonic stem cells [30]. On the other hand, many p53 binding proteins have been indentified in modulating the activity of p53 and p53-mediated apoptosis, such as the ASPP family members ASPP1, ASPP2 and the DNA binding protein YB1 [15, 16]. Nevertheless, the relationship between C23 and p53 is still not completely understood. To fully define the molecular mechanisms of p53 activity regulation, the priority is to discover the underlying collaborators of p53.

In this study, C23 interacted with p53 via its GAR domain and suppressed the transcriptional activity of p53 under DNA damage drug treatment and hypoxia. The GAR domain deleted mutation of C23 lost the binding ability to p53, and subsequently failed to promote tumor cell proliferation. In support, the elevated expression of C23, but not the mutant form of C23 lacking the GAR domain, prominently enhanced tumor growth in xenograft mouse model. In addition, the function of C23 in cell proliferation and survival upon DNA damage was significantly disturbed when p53 expression was inhibited. Therefore, p53 is most likely a major downstream mediator of C23 to control cell proliferation and apoptotic cell death during tumor development.

In summary, our study suggests that C23 plays a critical role in the regulation of tumorigenesis via suppressing p53 activity, and C23 may serve as a novel therapeutic target for cancer therapy.

## MATERIALS AND METHODS

### Cell culture and reagents

HCT116, HepG2, A549, H1299, HeLa, MCF-7, U2OS, Mel-RM, Mel-CV and HEK293T cells were maintained in DMEM (Invitrogen, CA) supplemented with 10% fetal bovine serum (FBS) and 1% penicillin-streptomycin. All cells were cultured in a humidified incubator at 37°C and 5% CO2.

The following antibodies were used in this study: GFP (Life, A11122 and Clonetech 632381), Flag (Sigma, F3165), GAPDH (Cell Signaling, 5174S), C23 (Santa Cruz Biotechnology, SC-8031 and Cell Signaling, 14574S), Caspase3 (Stressgene, AAP-113) and p53 (ABGENT, AJ1573a and Santa Cruz Biotechnology, SC-126). Polybrene and Doxorubicin were purchased from Sigma.

### Establishment of stable cells

Lentiviral plasmids psin-flag-Puro, psin-flag-C23-Puro or psin-flag-C23ΔGAR -Puro were co-transfected with pspax2 and pmd2.g into HEK293T cells using Lipo3000 (Invitrogen). Viral supernatant was collected 48 h post transfection, filtered and added to HCT116 cells in the presence of 8μg/ml polybrene. The transduced cells were selected by 0.5μg/ml puromycin. Lentivirus harboring shRNA used for targeting C23 or p53 were performed using the similar procedure described above. The sequences used to knockdown C23 and p53 are as follows: C23: 5′-CCTTGGAAATCCG TCTAGTTA-3′ and 5′-CGGTGAAATTGATGGAAA TAA-3′; p53: 5′-CGGCGCACAGAGGAAGAGAAT-3′ and 5′-TCAGACCTATGGAAACTACTT-3.′

### Co-immunoprecipitation

HCT116 cells were lysed in immunoprecipitation lysis buffer (0.5%NP-40, 150mM NaCl, 20mM HEPES, pH 7.5, 2mM EDTA, 1.5mM MgCl2) supplemented with protease inhibitor cocktail for 40 min on ice. The cell lysates were incubated with indicated antibody-conjugated protein A/G beads for 4 h at 4°C. The immunoprecipitates were washed by ice-cold lysis buffer with 0.05% NP-40 and boiled in SDS sample buffer. The samples were subjected to Western blot analysis.

### Luciferase reporter assay

To determine the effect of C23 on p53 transcriptional activity, the fragment including three-consensus p53 binding sites was cloned into pGL3-Basic Vector (Promega). HCT116 cells were cotransfected with pGL3-3×p53-BS-LUC and Renilla by Lipofectamine 3000. Firefly and Renilla luciferase activity were measured 48 h after transfection by a Dual-Luciferase Reporter Assay System (Promega). Firefly luciferase enzyme activity was normalized to Renilla luciferase enzyme activity.

### RNA isolation and qRT-PCR

Total RNA was extracted using Trizol (Invitrogen). 500ng of RNA was used to synthesize cDNA using PrimeScriptTM RT reagent kit (TaKaRa) according to the manufacturer's instruction. RT-qPCR was performed using SYBR premix EX Taq and ROX (TaKaRa) and analyzed with Stratagene Mx3000p (Agilent Technologies, Santa Clara, CA, USA). The following primers were used in this study for p53 target genes. NOXA: Forward 5′- GGAGATGCCTGGGAAGAAG-3′, Reverse 5′- TGCCGGAAGTTCAGTTTGTC-3′; BAK1: Forward 5′- AGAGTTCCAGACCATGTTGC −3′, Reverse 5′- GTAGCCGAAGCCCAGAAG -3′; DRAM: Forward 5′- GGTGTCTTTAGTGCTTGGATTG -3′, Reverse 5′- GATGGACTGTAGGAGCGTG -3′; TIGAR: Forward 5′- GGAAGAGTGCCCTGTGTTTAC -3′, Reverse 5′- AGTTGCTTGGAGATCCTTGG -3′; PUMA: Forward 5′- CGACCTCAACGCACAGTAC -3′, Reverse 5′- CCTAATTGGGCTCCATCTCG -3′; BAX: Forward 5′- GACATGTTTTCTGACGGCAAC -3′, Reverse 5′- AAGTCCAATGTCCAGCCC -3′; P21: Forward 5′- TGTCACTGTCTTGTACCCTTG -3′, Reverse 5′- GGCGTTTGGAGTGGTAGAA -3′.

### Western blot analysis

Western Blot analysis was performed as described previously [39].

### Colony formation assay

Cells were plated at a density of 5000 cells per well on a six-well plate. The cell culture medium was refreshed twice a week and cells were allowed to grow for 3 weeks. Then the cells were washed with PBS and fixed with cold 70% methanol for 15min, followed by staining with 0.005%(m/v) crystal violet for 30 min at room temperature and washed with water gently.

### Xenograft mouse model

Cells were injected subcutaneously into each flank of nude mice (Shanghai SLAC Laboratory, n = 6 mice per group). Tumor volume was monitored by caliper measurements and calculated by the modified ellipsoidal formula: tumor volume=1/2 length× width once 10 days [40], and tumors were weighed at 50 days after transplantation. Studies on animals were conducted with approval from the Animal Research Ethics Committee of Anhui Medical University.

### Patient tissue samples

Cancer lesions and corresponding normal adjacent tissues from 3 paired Lung cancer, 3 paired Colorectal cancer, 6 paired Breast cancer and 5 paired Gastric carcinoma were collected from the First Affiliated Hospital of Anhui Medical University (Hefei, China).

### The caspase-glo 3/7 assay

The caspase3/7 activity was measured using the cell lysate with the caspase3/7 assay kit (Promega G8090) according to the manufacturer's instruction.The activity was normalized to the protein concentration.

### Statistical analysis

Statistical analysis was performed using Microsoft Excel software (Microsoft, USA) and GraphPad Prism (GraphPad Software, USA). Student's *t*-test used to assess differences between experimental groups. A *P*-value less than 0.05 was considered to be statistical significant.

## SUPPLEMENTARY FIGURES



## References

[1] Mongelard F, Bouvet P (2007). Nucleolin: a multiFACeTed protein. Trends Cell Biol.

[2] Srivastava M, Pollard HB (1999). Molecular dissection of nucleolin's role in growth and cell proliferation: new insights. Faseb J.

[3] Campisi J, di Fagagna FD (2007). Cellular senescence: when bad things happen to good cells. Nat Rev Mol Cell Bio.

[4] Vousden KH, Prives C (2009). Blinded by the Light: The Growing Complexity of p53. Cell.

[5] Jiang P, Du WJ, Mancuso A, Wellen KE, Yang XL (2013). Reciprocal regulation of p53 and malic enzymes modulates metabolism and senescence. Nature.

[6] Levine AJ (1997). p53 the cellular gatekeeper for growth and division. Cell.

[7] Prives C, Hall PA (1999). The p53 pathway. J Pathol.

[8] Vogelstein B, Lane D, Levine AJ (2000). Surfing the p53 network. Nature.

[9] Li T, Kon N, Jiang L, Tan M, Ludwig T, Zhao Y, Baer R, Gu W (2012). Tumor suppression in the absence of p53-mediated cell-cycle arrest apoptosis and senescence. Cell.

[10] Petitjean A, Achatz MI, Borresen-Dale AL, Hainaut P, Olivier M (2007). TP53 mutations in human cancers: functional selection and impact on cancer prognosis and outcomes. Oncogene.

[11] Kato S, Han SY, Liu W, Otsuka K, Shibata H, Kanamaru R, Ishioka C (2003). Understanding the function-structure and function-mutation relationships of p53 tumor suppressor protein by high-resolution missense mutation analysis. Proceedings of the National Academy of Sciences of the United States of America.

[12] Aylon Y, Oren M (2007). Living with p53 dying of p53. Cell.

[13] Jin L, Hu WL, Jiang CC, Wang JX, Han CC, Chu P, Zhang LJ, Thorne RF, Wilmott J, Scolyer RA, Hersey P, Zhang XD, Wu M (2011). MicroRNA-149* a p53-responsive microRNA functions as an oncogenic regulator in human melanoma. Proceedings of the National Academy of Sciences of the United States of America.

[14] Kruse JP, Gu W (2009). Modes of p53 regulation. Cell.

[15] Sullivan A, Lu X (2007). ASPP: a new family of oncogenes and tumour suppressor genes. Brit J Cancer.

[16] Homer C, Knight DA, Hananeia L, Sheard P, Risk J, Lasham A, Royds JA, Braithwaite AW (2005). Y-box factor YB1 controls p53 apoptotic function. Oncogene.

[17] Laptenko O, Prives C (2006). Transcriptional regulation by p53: one protein many possibilities. Cell death and differentiation.

[18] Ginisty H, Sicard H, Roger B, Bouvet P (1999). Structure and functions of nucleolin. J Cell Sci.

[19] Srivastava M, Pollard HB (1999). Molecular dissection of nucleolin's role in growth and cell proliferation: new insights. Faseb J.

[20] Greasley PJ, Bonnard C, Amati B (2000). Myc induces the nucleolin and BN51 genes: possible implications in ribosome biogenesis. Nucleic acids research.

[21] Sengupta TK, Bandyopadhyay S, Fernandes DJ, Spicer EK (2004). Identification of nucleolin as an AU-rich element binding protein involved in bcl-2 mRNA stabilization. Journal of Biological Chemistry.

[22] Zheng X, Zhang Y, Chen YQ, Castranova V, Shi X, Chen F (2005). Inhibition of NF-kappaB stabilizes gadd45alpha mRNA. Biochem Biophys Res Commun.

[23] Iftode C, Daniely Y, Borowiec JA (1999). Replication protein A (RPA): the eukaryotic SSB. Critical reviews in biochemistry and molecular biology.

[24] Daniely Y, Borowiec JA (2000). Formation of a complex between nucleolin and replication protein a after cell stress prevents initiation of DNA replication. Journal of Cell Biology.

[25] De A, Donahue SL, Tabah A, Castro NE, Mraz N, Cruise JL, Campbell C (2006). A novel interaction [corrected] of nucleolin with Rad51. Biochem Biophys Res Commun.

[26] Wu YL, Dudognon C, Nguyen E, Hillion J, Pendino F, Tarkanyi I, Aradi J, Lanotte M, Tong JH, Chen GQ, Segal-Bendirdjian E (2006). Immunodetection of human telomerase reverse-transcriptase (hTERT) re-appraised: nucleolin and telomerase cross paths. J Cell Sci.

[27] Takagi M, Absalon MJ, McLure KG, Kastan MB (2005). Regulation of p53 translation and induction after DNA damage by ribosomal protein L26 and nucleolin. Cell.

[28] Daniely Y, Dimitrova DD, Borowiec JA (2002). Stress-dependent nucleolin mobilization mediated by p53-nucleolin complex formation. Molecular and cellular biology.

[29] Saxena A, Rorie CJ, Dimitrov D, Daniely Y, Borowiec JA (2006). Nucleolin inhibits Hdm2 by multiple pathways leading to p53 stabilization. Oncogene.

[30] Yang A, Shi GL, Zhou CL, Lu R, Li H, Sun L, Jin Y (2011). Nucleolin Maintains Embryonic Stem Cell Self-renewal by Suppression of p53 Protein-dependent Pathway. Journal of Biological Chemistry.

[31] Bhatt P, d'Avout C, Kane NS, Borowiec JA, Saxena A (2012). Specific domains of nucleolin interact with Hdm2 and antagonize Hdm2-mediated p53 ubiquitination. The FEBS journal.

[32] Kastan MB (2007). Wild-type p53: tumors can't stand it. Cell.

[33] Fuster JJ, Sanz-Gonzalez SM, Moll UM, Andres V (2007). Classic and novel roles of p53: prospects for anticancer therapy. Trends in molecular medicine.

[34] Brady CA, Jiang D, Mello SS, Johnson TM, Jarvis LA, Kozak MM, Kenzelmann Broz D, Basak S, Park EJ, McLaughlin ME, Karnezis AN, Attardi LD (2011). Distinct p53 transcriptional programs dictate acute DNA-damage responses and tumor suppression. Cell.

[35] Tuteja R, Tuteja N (1998). Nucleolin: a multifunctional major nucleolar phosphoprotein. Critical reviews in biochemistry and molecular biology.

[36] Berger CM, Gaume X, Bouvet P (2015). The roles of nucleolin subcellular localization in cancer. Biochimie.

[37] Abdelmohsen K, Gorospe M (2012). RNA-binding protein nucleolin in disease. Rna Biol.

[38] Joo EJ, Yang H, Park Y, Park NY, Toida T, Linhardt RJ, Kim YS (2010). Induction of Nucleolin Translocation by Acharan Sulfate in A549 Human Lung Adenocarcinoma. J Cell Biochem.

[39] Hu W, Jin L, Jiang CC, Long GV, Scolyer RA, Wu Q, Zhang XD, Mei Y, Wu M (2013). AEBP1 upregulation confers acquired resistance to BRAF (V600E) inhibition in melanoma. Cell Death Dis.

[40] Prahallad A, Sun C, Huang S, Di Nicolantonio F, Salazar R, Zecchin D, Beijersbergen RL, Bardelli A, Bernards R (2012). Unresponsiveness of colon cancer to BRAF(V600E) inhibition through feedback activation of EGFR. Nature.

