# Supplementary material for: C23 promotes tumorigenesis via suppressing p53 activity
Source: Oncotarget. 2016 Aug 5;7(36):58274–85. doi: 10.18632/oncotarget.11071 (PMC5295430; doi:10.18632/oncotarget.11071)
Supplement: Supplementary file 1 [file oncotarget-07-58274-s001.pdf]

C23 promotes tumorigenesis via suppressing p53 activity

SUPPLEMENTARY FIGURES

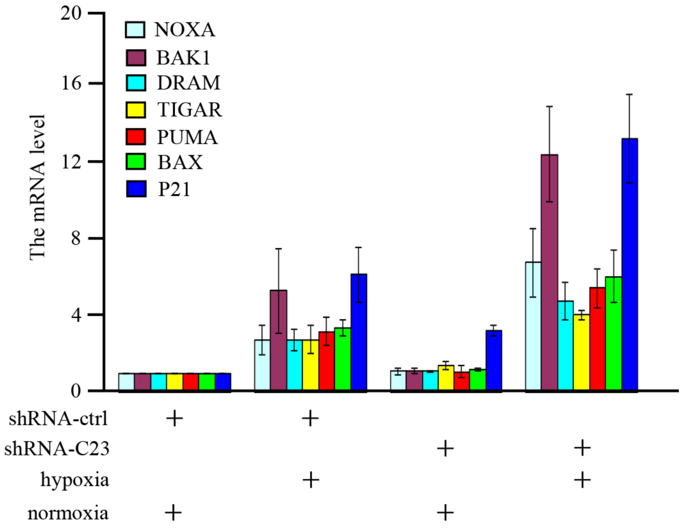

Supplementary Figure S1: HCT116 cells with and without stable knockdown of C23 were cultured under normoxic (20% O_2) or hypoxic (1% O_2) condition for 24 hours, the p53 target gene mRNA expression levels were then analyzed by qRT-PCR analysis.

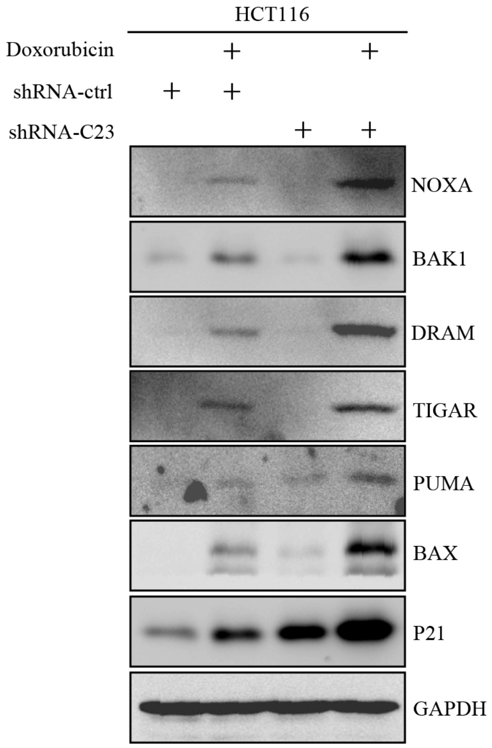

Supplementary Figure S2: HCT116 cells with and without stable knockdown of C23 were under Doxorubicin (100ng/ml) treatment for 24 hours, then the protein level of p53 target genes were analyzed by western blot analysis.

a

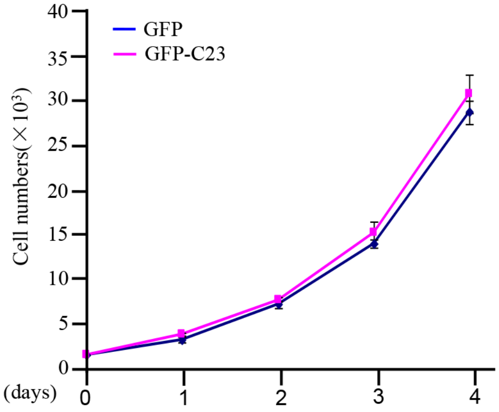

b

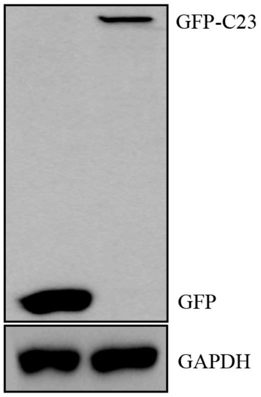

Supplementary Figure S3: a. The number of H1299 cells with GFP or GFP-C23 transfection were analyzed by cell counter. The data were represented as mean±S.D. of three independent experiments. **b.** The expression of GFP and GFP-C23 in H1299 cells were analyzed with anti-GFP antibody.

a

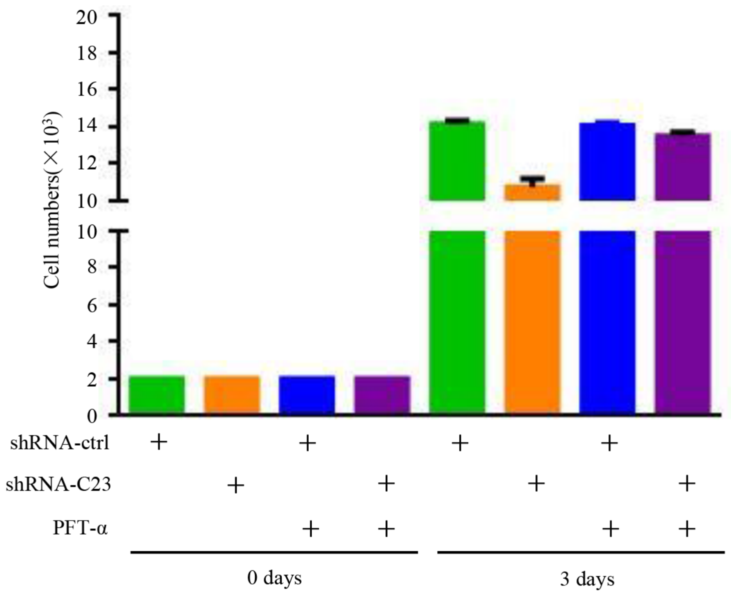

b

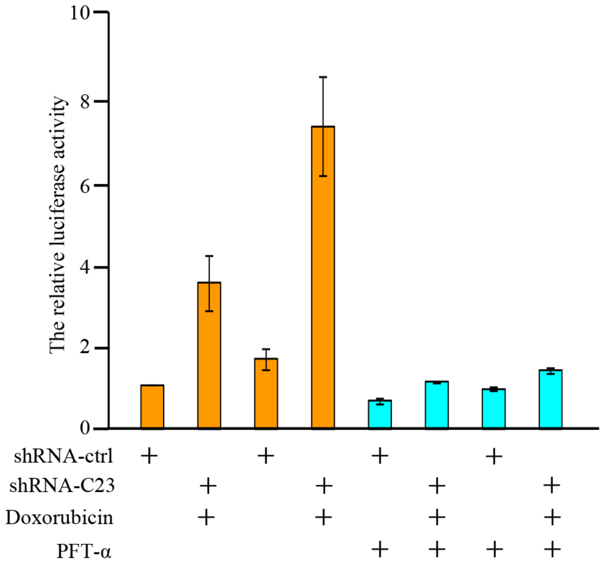

Supplementary Figure S4: a. HCT116 cells with and without stable knockdown of C23 were treated with 20uM pifithrin-alpha for 3 days. The cell numbers were analyzed by cell counter. The data were represented as mean±S.D. of three independent experiments. **b.** HCT116 cells with and without C23 shRNA were co-transfected with pGL3-3×p53-BS-LUC and Renilla. 24 hours after transfection, cells were treated with Doxorubicin (100ng/ml) or combined with pifithrin-alpha (20uM) for 24 hours. The cell lysates were then analyzed by lucifease assay.
